# Heterogeneity of benefit finding in maintenance hemodialysis patients: a decision tree-based subgroup analysis of self-efficacy and social support

**DOI:** 10.3389/fpsyt.2025.1665458

**Published:** 2025-09-18

**Authors:** Yan Fan, Tianci Tong, Yiru Wang, Yanlin Gong, Jing Wu, Jing Chu

**Affiliations:** ^1^ School of Nursing, Naval Medical University, Shanghai, China; ^2^ Department of Clinical Psychology, Chongqing Mental Health Center, Chongqing, Shanghai, China

**Keywords:** renal dialysis, self-efficacy, social support, benefit finding, decision tree

## Abstract

**Background:**

Benefit finding (BF) improves quality of life in maintenance hemodialysis (MHD) patients, yet population heterogeneity remains understudied. This study explores how self-efficacy and social support jointly influence BF patterns and identifies distinct patient subgroups.

**Methods:**

This multi-center cross-sectional study was conducted from April to September 2023 at five tertiary hospitals in Shanghai, China, enrolling 352 MHD patients. Data from the Benefit Finding Scale, General Self-Efficacy Scale, and Perceived Social Support Scale were used to construct a Classification and Regression Tree (CART) model employing five-fold cross-validation, with a maximum depth of 3 and a minimum leaf node size of 10%.

**Results:**

The CART model (R²=0.278) identified five distinct BF subgroups (*p*<0.001): Low Self-Efficacy Constrained Group, Psychological Resource Deficient Group, Internally Belief Driven Group, Balanced Resource Adaptation Group, and Resource Integrated Advantage Group, each characterized by unique combinations of self-efficacy and social support. Significant differences were observed among the subgroups in terms of gender(*p*=0.045), education level(*p*=0.010), and employment status(*p*=0.003).

**Conclusion:**

BF levels in MHD patients demonstrated significant variations influenced by the combined effects of self-efficacy and social support. The decision tree model successfully identified patient subgroups with distinct psychological resource configurations. These findings provide a theoretical foundation for implementing stratified and personalized psychological interventions in clinical practice. Clinicians can identify and prioritize vulnerable patients who simultaneously lack self-efficacy and social support, offering them targeted positive psychological interventions that may potentially improve treatment adherence and long-term prognosis.

## 1 Introduction

Chronic kidney disease (CKD) has emerged as a significant global public health concern, affecting approximately 9.5% of the world’s population (1). In China, CKD affects 130 million individuals (2). When glomerular filtration rate (GFR) declines to below 15 mL/min/1.73m² or dialysis begins, the condition progresses to stage five CKD, known as end-stage renal disease (ESRD) (3). Maintenance hemodialysis (MHD) represents the primary renal replacement therapy for ESRD patients (4). According to Fresenius Medical Care’s annual report, approximately 3.63 million patients worldwide receive hemodialysis, with China having the largest dialysis population exceeding 916,000 patients (5). While MHD significantly extends survival, it introduces multiple challenges for patients. Physiologically, 44.2-73.5% of patients experience pruritus (6), up to 97% face fatigue issues (7). These physiological symptoms further exacerbate psychological burden, with depression and anxiety prevalence ranging from 13.1% to 76.3% among MHD patients, significantly affecting cognitive function (8, 9), treatment adherence, social participation, and quality of life (10–12). Therefore, exploring effective strategies to enhance psychological adaptation in MHD patients has substantial clinical significance.

Benefit finding (BF), a core concept in positive psychology derived from Taylor’s Cognitive Adaptation Theory, refers to an individual’s perception of positive psychological and behavioral changes when facing major adverse life events (13). International research has documented this phenomenon across various chronic conditions, including cancer, diabetes, and pulmonary diseases (14, 15). Studies confirm that patients with higher BF levels typically demonstrate better psychological adjustment, higher treatment adherence, and superior quality of life (16–18).

However, BF exhibits significant heterogeneity among chronic disease patients. Research indicates that BF levels vary according to social environment, and individual characteristics (19). Longitudinal studies further reveal that BF evolves dynamically over time, with psychosocial factors often demonstrating stronger explanatory power than medical characteristics in predicting this process (20). These findings collectively point to a conclusion: complex interactions between psychological resources likely constitute the core source of BF variability (21), with self-efficacy and social support emerging as particularly influential factors (22). Our preliminary research powerfully confirmed the decisive role of these two factors in promoting BF among MHD patients (22, 23). Cross-sectional analysis demonstrated that, after controlling for confounding factors, perceived social support was significantly positively correlated with BF in MHD patients (p<0.001). Longitudinal investigation through multinomial logistic regression further revealed that general self-efficacy and perceived social support were the strongest predictors of BF trajectory changes, especially in the high-rising group, with Wald values (21.406 and 16.319, respectively) significantly higher than other predictive variables, highlighting their central role in the formation and development of BF among MHD patients.

According to the Conservation of Resources Theory proposed by Hobfoll, psychological adaptation essentially involves the acquisition, preservation, and allocation of resources (24). BF can be conceptualized as a positive psychological adaptation outcome for long-term dialysis patients facing disease challenges, while self-efficacy and social support function as key predictors facilitating this outcome. These psychological resources operate through specific mechanisms in promoting BF, influencing outcomes independently and reinforcing each other. Individuals with high self-efficacy better identify and utilize available social support, while adequate social support enhances self-efficacy through providing successful experiences and positive feedback, creating a virtuous cycle of resource accumulation that promotes positive psychological transformation (25).

Self-efficacy, a core concept in Social Cognitive Theory, reflects an individual’s confidence in their ability to execute specific behaviors or achieve desired goals (15). In the context of maintenance hemodialysis, it manifests as patients’ perceived control and willingness to adhere to health-promoting behaviors. Research indicates that MHD patients generally demonstrate moderate levels of self-efficacy (26). As a critical psychological resource, self-efficacy effectively regulates psychological adaptation processes, enhances coping capabilities among chronic disease patients, and accounts for approximately 28% of the variance in health behaviors and psychological adjustment outcomes (27). Patients with high self-efficacy are more likely to actively engage in treatment protocols, initiate self-management practices, and maintain psychological resilience, serving as a fundamental internal resource for promoting BF development (28, 29).

Social support refers to the emotional, informational, and practical assistance individuals receive from their social networks (30). For MHD patients, primary support sources include care from close family members, professional communication with healthcare providers, and mutual assistance among fellow patients. Research indicates moderate overall support levels among these patients (31). While the relative importance of different support sources may vary across cultural contexts, adequate social support consistently correlates with positive psychological outcomes (32). Stable social support can enhance a sense of security, improve psychological adaptation, and lay the foundation for the reconstruction of meaning and the discovery of benefits (33).

To investigate how different configurations of self-efficacy and social support shape BF levels and their potential heterogeneity, this study employed the Classification and Regression Tree (CART) model, a machine learning approach, to examine how these psychological resources interact to form distinct patterns of perceived benefits among MHD patients. The CART model offers unique advantages for understanding BF heterogeneity through its ability to identify non-linear relationships, capture complex interaction effects, and create clinically meaningful patient subgroups (34).

This study aims to: (1) explore the heterogeneity of BF among MHD patients using a regression-based decision tree model with self-efficacy and social support as stratification variables; (2) analyze demographic and clinical characteristic differences across identified subgroups; and (3) provide theoretical foundation and practical guidance for developing targeted, stratified psychological interventions.

## 2 Methods

### 2.1 Study design

This study employed a multicenter cross-sectional survey design, aiming to identify distinct subgroups of BF among MHD patients and to provide a foundation for implementing targeted psychosocial interventions.

### 2.2 Participants

This study recruited 364 MHD patients from the nephrology inpatient departments of five tertiary general hospitals in Shanghai, China, between April and September 2023.

Inclusion criteria were as follows:(1) Diagnosed with ESRD (32); (2) Aged≥18 years; (3) Undergoing regular hemodialysis treatment for at least 3 months; (4) Clear consciousness, adequate communication ability, and voluntary participation in the study. Exclusion criteria included: (1)Patients with psychiatric disorders, cognitive impairment, or inability to complete the survey as confirmed by medical records or self-report(2) Severe complications or dysfunction of major organs such as the heart or liver.

During data quality control, 12 questionnaires were excluded due to incomplete responses or errors, resulting in 352 valid questionnaires, with an effective response rate of 96.70%.

The sample size was determined based on the empirical rule for CART analysis. Considering that this study included two primary predictor variables (self-efficacy and social support), each potentially generating two to three meaningful branches, and accounting for possible interaction effects, we anticipated approximately eight terminal nodes in the final model. Following the rule that each terminal node should contain at least 30 observations, the minimum required sample size was estimated to be 240 participants. Our final sample of 352 valid participants exceeded this requirement, ensuring the statistical stability of the analysis (35).

### 2.3 Measurement instruments

#### 2.3.1 Demographic and clinical data questionnaire

The questionnaire was developed by the research team based on a review of the literature and included information on demographic variables such as gender, age, marital status, and employment status. It also collected clinical data including disease etiology, presence of chronic co-morbidities, duration of dialysis, complications associated with haemodialysis, etc.

#### 2.3.2 Benefit finding scale

The BFS was developed by our research team based on literature review, qualitative interviews, and quantitative analysis. It comprises 26 items across six dimensions—personal growth, health behavior change, appreciation of life, realization of social support, altruism, and spiritual development—rated on a 5-point Likert scale (0 = “not at all” to 4 = “very much”). Total scores range from 0 to 104, with higher scores indicating greater BF. The BFS demonstrated strong psychometric properties following content validity assessment, exploratory factor analysis, and cultural adaptation, with an overall Cronbach’s α of 0.924 and subscale coefficients of 0.66–0.89 (36). Full item listings and psychometric details are provided in the online **Supplementary Materials**.

#### 2.3.3 General self-efficacy scale

Originally developed by Professor Ralf Schwarzer and colleagues in 1981 (37), the GSES contains 10 items rated on a 4-point Likert scale from “Not at all true” (1) to “Exactly true” (4). Total scores range from 10 to 40, with higher scores reflecting greater confidence in one’s ability to cope with challenging demands. The scale has demonstrated good reliability with a Cronbach’s α of 0.87.

#### 2.3.4 Perceived social support scale

Developed by Blumenthal and colleagues and later translated and adapted into Chinese by domestic scholars (38), the PSSS measures perceived support from three sources: family, friends, and significant others. It includes 12 items (four per subscale), rated on a 7-point Likert scale from “Very strongly disagree” (1) to “Very strongly agree” (7). Total scores range from 12 to 84, with higher scores indicating greater perceived social support. The scale showed good internal consistency, with a Cronbach’s α of 0.84.

### 2.4 Data collection

Data were collected by uniformly trained researchers during patients’ hemodialysis sessions. Eligible patients were informed of the study’s objectives and procedures, and written informed consent was obtained prior to participation. Participants were then guided to complete the questionnaire independently. For those with reading difficulties, researchers read the items in a standardized manner and recorded the responses. Upon completion, each questionnaire was immediately reviewed for completeness, and any with missing or invalid responses were excluded from the dataset. The entire process required approximately 15–20 minutes per participant.

### 2.5 Statistical analysis

All statistical analyses were conducted using Python 3.12 and IBM SPSS version 27.0. Python-based analyses employed data processing and visualization libraries such as pandas, numpy, scikit-learn, and matplotlib.

Categorical demographic variables were described using frequencies and percentages [n(%)]. Continuous variables were tested for normality using the Shapiro-Wilk test. Data conforming to normal distribution were reported as mean ± standard deviation (Mean ± SD), while non-normally distributed data were described using the median and interquartile range (IQR).

To explore the interaction between self-efficacy and social support in relation to BF and to identify patient subgroups, a regression-based decision tree model was employed. The total BF score served as the target variable, with self-efficacy and social support scores as predictor variables. For model parameter settings, a maximum tree depth was 3 to ensure interpretability of the results while avoiding overfitting (39). The minimum leaf node size was set to 10% of the total sample to ensure that each subgroup contained sufficient observations for reliable statistical inference (35). To evaluate the model’s stability and generalizability, a five-fold cross-validation approach was applied.This cross-validation strategy was chosen based on our sample size (n = 352), as it provides an optimal balance between reliable model assessment and computational efficiency. The Coefficient of Determination (R²), Mean Squared Error (MSE), and Mean Absolute Error (MAE) were calculated for each fold to assess predictive performance and stability (40).

Based on the data distribution, the chi-square test or Fisher’s exact test was applied for categorical variables, and the Kruskal–Wallis H test or one-way analysis of variance (ANOVA) was used for continuous variables to examine subgroup differences in demographic and clinical characteristics. For variables showing significant differences (*p* < 0.05), *post hoc* multiple comparisons were conducted using the Bonferroni correction to control for inflation of type I error. A p-value < 0.05 was considered statistically significant.

## 3 Results

### 3.1 Demographic characteristics

A total of 352 patients undergoing MHD were enrolled in this study, including 193 males (54.83%) and 159 females (45.17%). Among them, 152 patients (43.18%) were aged over 60 years, with a mean duration dialysis of 7.51 ± 6.78 years. Additional demographic characteristics of the participants are detailed in **Table 1**.

**Table 1:** Demographic characteristics of MHD patients (N=352).

Variable	*N (%)*
Gender	
Male	193 (54.83)
Female	159 (45.17)
Age	
18-30	4 (1.1)
31-40	42 (11.9)
41-50	77 (21.88)
51-60	77 (21.88)
>60	152 (43.18)
Marital status	
Married	267 (75.85)
Unmarried	48 (13.64)
Widowed	25 (7.10)
Divorced	12 (3.41)
Living arrangement	
Living alone	28 (7.95)
Living with family	321 (91.19)
Other	3 (0.85)
Primary caregiver	
Self	153 (43.47)
Family member	189 (53.69)
Other	10 (2.84)
Education level	
Primary school or below	11 (3.13)
Junior high school	76 (21.59)
Senior high school	128 (36.36)
College	125 (35.51)
Master’s degree or above	12 (3.41)
Employment status	
Full-time employed	69 (19.60)
Resigned	19 (5.40)
Retired/Pensioned	212 (60.23)
Unemployed	52 (14.77)
Healthcare payment method	
Employee Medical Insurance	273 (77.56)
Urban Resident Medical Insurance	64 (18.18)
Rural Cooperative Medical Scheme	5 (1.42)
Commercial Insurance	4 (1.14)
Other	66 (1.70)
Financial burden of healthcare costs	
Very mild	30 (8.52)
Mild	124 (35.23)
Moderate	115 (32.67)
Severe	61 (17.33)
Very severe	22 (6.25)
Primary etiology of kidney disease	
Chronic Glomerulonephritis	113 (32.10)
Diabetic Nephropathy	45 (12.78)
Hypertensive Nephropathy	66 (18.75)
Polycystic Kidney Disease	2 (0.57)
Hereditary Nephropathy	44 (12.50)
Idiopathic	44 (12.50)
Other	38 (10.80)
Other chronic kidney diseases	
Yes	180 (51.14)
No	172 (48.86)
Duration of hemodialysis	M=7.51 (SE=6.78)
Hemodialysis complications	
Yes	99 (28.13)
No	253 (71.88)
History of kidney transplantation	
Yes	31 (8.81)
No	321 (91.19)
Kidney transplant willingness	
None	169 (48.01)
Moderate	125 (35.51)
Strong	58 (16.48)
Self-perceived disease severity	
Very mild	5 (1.42)
Mild	54 (15.34)
Moderate	182 (51.70)
Severe	89 (25.28)
Very severe	22 (6.25)
Knowledge level of hemodialysis	
Very familiar	28 (7.95)
Fairly familiar	133 (37.78)
Partially familiar	136 (38.64)
Slightly familiar	48 (13.64)
Not familiar at all	7 (1.99)

M, Mean; SE, Standard Error.

### 3.2 BFS, GSE, and PSSS scores

Among the 352 MHD patients, the BFS, GES and PSSS scores were median (IQR) 63.00 (21.00), 24.50 (9.00) and 56.00 (16.00) respectively, suggesting a moderate to high level of overall psychological resources, **Table 2** presents the detailed distribution of scores for the scale and its individual dimensions.

**Table 2:** Scores of BFS, GES, and PSSS.

Viariable	Item	Scores [M, IQR]	Scale Range
BF dimensions			
Spiritual growth	2	4.00 (2)	0-8
Appreciation of living and life	5	14.00 (4)	0-20
Awareness of social support	4	10.00 (4)	0-16
Personal growth	7	17.00 (7)	0-28
Altruistic behavior	3	7.00 (5)	0-12
Health behavior changes	5	12.00 (5)	0-20
BFS	26	63.00 (21)	0-104
GSE	10	24.50 (9)	10-40
Perceived social support dimensions			
Family support	4	23.00 (5)	4-28
Friend support	4	17.00 (7)	4-28
Other support	4	17.00 (6.75)	4-28
PSSS	12	56.00 (16)	12-84

M, median; IQR, interquartile range.

M, Mean; SE, Standard Error.

### 3.3 Performance evaluation of the CART

A five-fold cross-validation was conducted to assess the performance and stability of the decision tree model. The cross-validation results showed a mean coefficient of determination (R²) of 0.126 (SD = 0.068), a total R² of 0.278, a mean squared error (MSE) of 212.204, and a mean absolute error (MAE) of 11.203. These findings indicate that the final model explained 27.8% of the variance in BF, with an average prediction error of approximately 11.2 points. This level of predictive accuracy provides meaningful information for identifying subgroup differences in BF.

### 3.4 Subgroup classification of BF based on decision tree model

**Figure 1:**
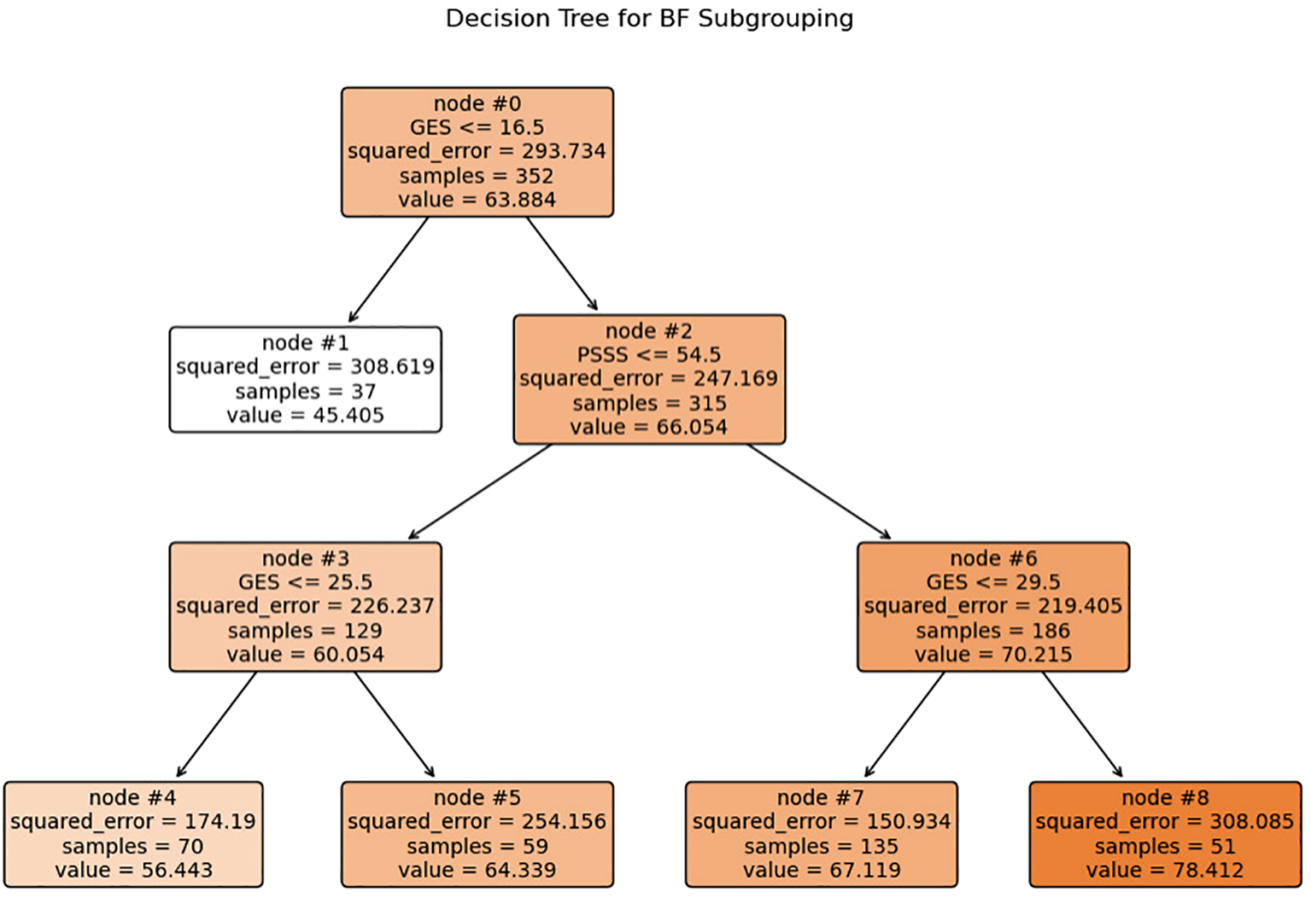
Decision tree model identifying subgroups of BF based on self-efficacy and social support. Each internal node shows the splitting criterion (e.g.”GSES≤ 16.5”) with the variable name and threshold value. Terminal nodes (leaves) are labeled 1, 4, 5, 7, and 8, representing the five distinct patient subgroups. Values inside each node indicate: squared_error, samples, and value (mean BF score). The color intensity corresponds to the mean BF score, with darker colors indicating higher BF levels. GSES, General Self-Efficacy Scale score; PSSS, Perceived Social Support Scale score.

**Figure 2:**
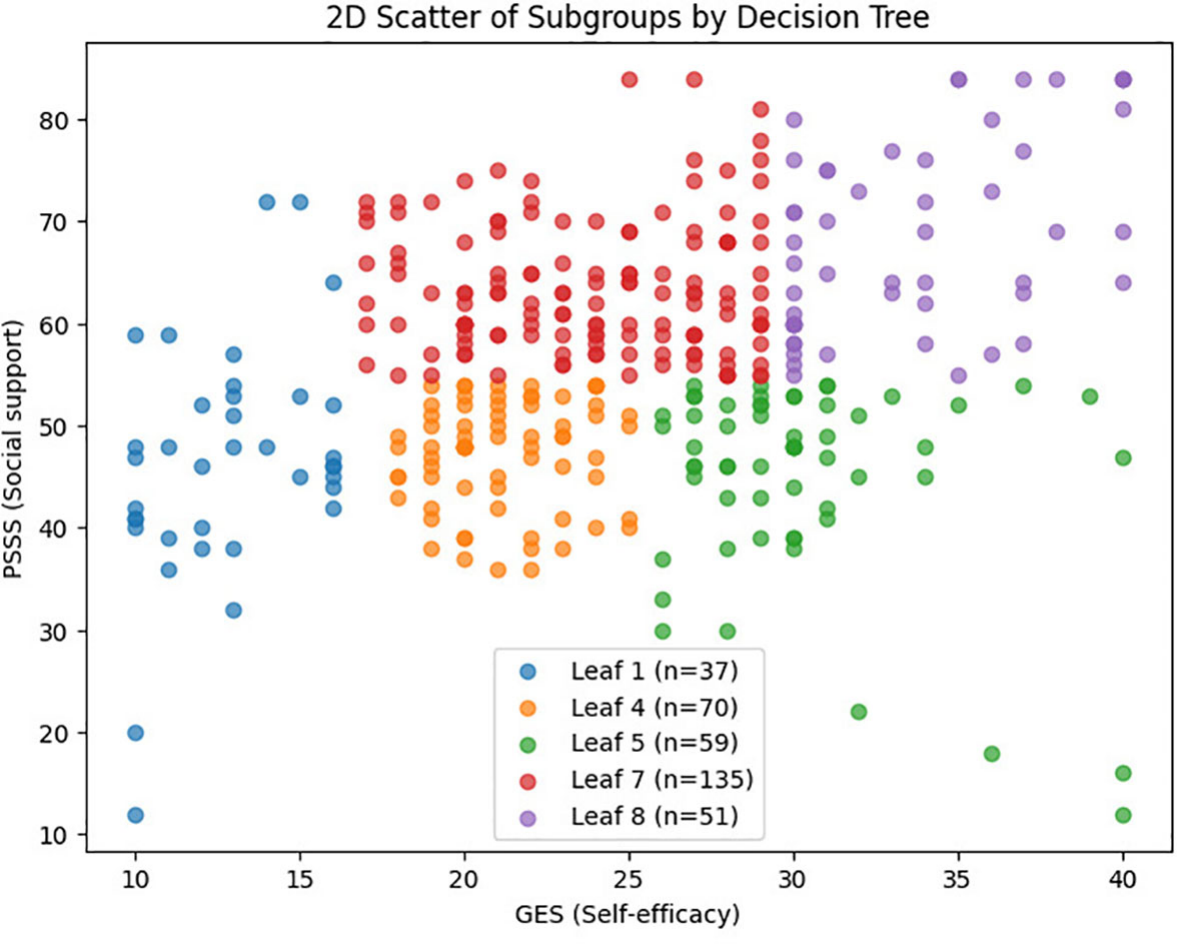
2D scatter plot of BF subgroups based on self-efficacy and social support. **Figure 2** showing the distribution of patients across the five BF subgroups based on self-efficacy (x-axis) and social support (y-axis) scores. Each dot represents an individual patient, with colors indicating subgroup membership: Low Self-Efficacy Constrained Group (red), Psychological Resource Relatively Deficient Group (orange), Internally Belief Driven Group (green), Balanced Resource Adaptation Group (blue), and Psychological Resource Integrated Advantage Group (purple).

**Figure 3:**
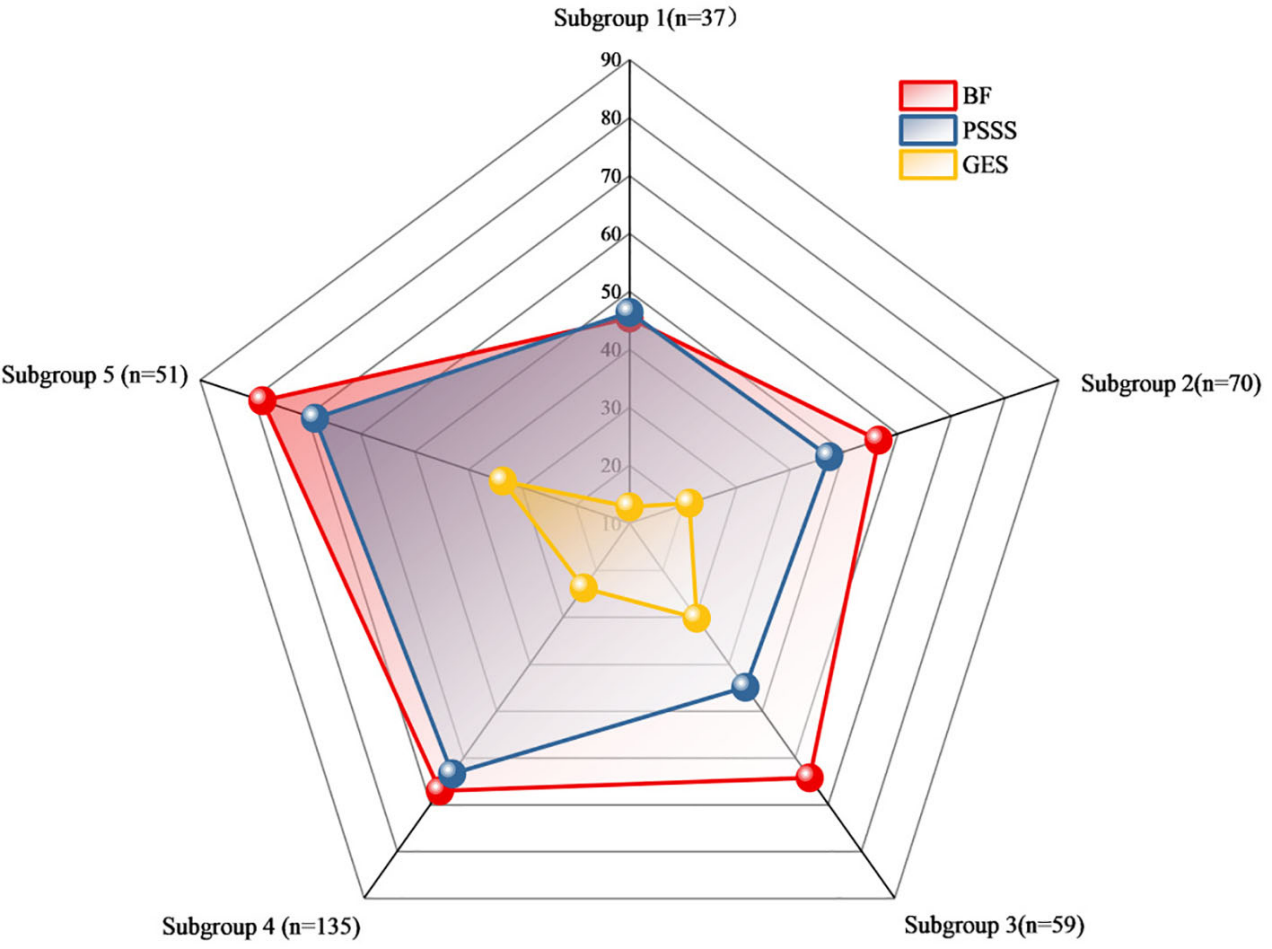
Radar chart of the subgroup analysis of benefits based on self-efficacy and social support. **Figure 3** Radar chart comparing mean scores of BFS, PSSS and GSES among the five identified subgroups. Each axis represents one of the five subgroups: Subgroup 1 (n=37, Low Self-Efficacy Constrained Group), Subgroup 2 (n=70, Psychological Resource Relatively Deficient Group), Subgroup 3 (n=59, Internally Belief Driven Group), Subgroup 4 (n=135, Balanced Resource Adaptation Group), and Subgroup 5 (n=51, Psychological Resource Integrated Advantage Group). The scale (0-90) represents the mean scores for each measure within each subgroup. The red line shows mean BF scores, blue line shows mean PSSS scores, and yellow line shows mean GES scores for each subgroup.

Based on a CART model, five distinct subgroups of BF were identified among MHD patients, using social support and self-efficacy as the splitting variables (**Figures 1**–**3**, **Table 3**).

**Table 3:** Characteristics of BF subgroups identified by decision tree.

Group	Sample size (n,%)	GSE score	PSSS score	BF score	Group designation
1	37 (10.51%)	≤16.5	12-72	45.41	Low Self-Efficacy Constrained Group
2	70 (19.89%)	16.5<GES ≤ 25.5	≤54.5	56.44	Psychological Resource Relatively Deficient Group
3	59 (16.76%)	>25.5	≤54.5	64.34	Internally Belief Driven Group
4	135 (38.35%)	≤29.5	>54.5	67.12	Balanced Resource Adaptation Group
5	51 (14.48%)	>29.5	>54.5	78.41	Psychological Resource Integrated Advantage Group

In the first subgroup, the social support variable was not selected as a splitting node in the model, but its actual score ranged from 12 to 72 and was only shown as a feature without splitting efficacy.

Subgroup1 (leaf1, n=37, 10.51%) –Low Self-Efficacy Constrained Group: Characterized by markedly low self-efficacy (GES ≤16.5), with minimal internal coping capacity and the lowest BF score (M = 45.41). Social support did not contribute significantly to subgroup differentiation.

Subgroup2 (leaf4, n=70, 19.89%) – Psychological Resource Relatively Deficient Group: Both self-efficacy (16.5 < GES ≤25.5) and social support (PSSS ≤54.5) were below average. Limited psychological resources corresponded to a modest BF score (M = 56.44).

Subgroup 3 (leaf5, n=59, 16.76%) – Internally Belief Driven Group: Despite low social support (PSSS ≤54.5), higher self-efficacy (GES >25.5) supported a relatively favorable BF level (M = 64.33), highlighting the compensatory role of internal resources.

Subgroup4 (leaf7, n=135, 38.35%) –Balanced Resource Adaptation Group: Patients showed moderately high levels of both self-efficacy (25.5 < GES ≤29.5) and social support (PSSS >54.5), achieving above-average BF (M = 67.12) through balanced resource utilization.

Subgroup5 (leaf8, n=51, 14.48%)–Psychological Resource-Integrated Advantage Group: With the highest levels of both self-efficacy (GES >29.5) and social support (PSSS >54.5), this group exhibited the greatest BF (M = 78.41), reflecting optimal psychological resilience and adaptation.

All categorical variables showed statistically significant differences across the five subgroups (p < 0.001). *Post hoc* multiple comparisons with Bonferroni correction revealed significant differences between specific subgroup pairs. Detailed subgroup scores and *post hoc* comparison results are presented in **Table 4**.

**Table 4:** Comparison of scores among the five subgroups of self-efficacy, social support, and benefit discovery.

Variable	Subgroup 1 (n=37)	Subgroup 2 (n=70)	Subgroup 3 (n=59)	Subgroup 4 (n=135)	Subgroup 5 (n=51)	± S	F-value	p
BF	45.41± 17.81^cde^	56.44± 13.29^de^	64.33± 16.07^ae^	67.12± 12.33^abe^	78.4 ± 17.73^abcd^	63.88 ± 17.16	33.33	<0.001
GES	12.81 ± 2.27^bcde^	21.17 ± 1.99^acde^	30.25 ± 3.56^abde^	23.80 ± 3.70^abce^	33.60 ± 3.53^abcd^	24.62 ± 6.69	286.03	<0.001
PSSS	46.40 ± 11.63^de^	47.21± 5.28^de^	44.98± 9.71^de^	63.44 ± 6.44^abce^	68.58 ± 9.43^abcd^	56.09 ± 12.39	123.07	<0.001

Data are presented as mean ± standard deviation. Superscript letters indicate significant between-group differences after Bonferroni correction (P < 0.05). a: vs. Subgroup 1, P < 0.05; b: vs. Subgroup 2, P < 0.05; c: vs. Subgroup 3, P < 0.05; d: vs. Subgroup 4, P < 0.05; e: vs. Subgroup 5, P < 0.05. All *p-*values were adjusted using Bonferroni correction to control for type I error in multiple comparisons.

### 3.5 Comparison of demographic and baseline characteristics across subgroup

Statistically significant differences were observed across subgroups in terms of gender, educational level, and employment status (*p* < 0.05), suggesting an association between psychological resource profiles and sociodemographic characteristics. The detailed comparative analysis of demographic and baseline characteristics among subgroups is presented in **Table 5**.

**Table 5:** Demographic comparison among BF subgroups.

Variable	Subgroup 1 (n=37)	Subgroup 2 (n=70)	Subgroup 3 (n=59)	Subgroup 4 (n=135)	Subgroup 5 (n=51)	χ²/F/H	*p*
Gender						χ2 = 9.722	0.045
Male	17 (45.94)	39 (55.71)	39 (66.10)	78 (57.77)	20 (39.21)		
Female	20 (54.05)	31 (44.29)	20 (33.90)	57 (42.22)	31 (60.78)		
Age						F=17.120	0.316
18-30	0 (.00)	1 (1.43)	0 (.00)	2 (1.48)	1 (1.96)		
31-40	2 (5.41)	6.00 (8.57)	11 (18.64)	14 (10.37)	9 (17.65)		
41-50	6 (16.22)	12 (17.14)	13 (22.03)	30.00 (22.22)	16 (31.37)		
51-60	6 (16.22)	19 (27.14)	11 (18.64)	32 (23.70)	9 (17.65)		
>60	23 (62.16)	32 (45.71)	24 (40.68)	57 (42.22)	16 (31.37)		
Marital status						F=14.581	0.277
Married	27 (72.97)	59 (84.29)	39 (66.10)	103 (76.30)	39 (76.47)		
Unmarried	3 (8.11)	8 (11.43)	11 (18.64)	16 (11.85)	10 (19.61)		
Widowed	5 (13.51)	2 (2.86)	7 (11.86)	10 (7.41)	1 (1.96)		
Divorced	2.00 (5.41)	1 (1.43)	2 (3.39)	6 (4.44)	1 (1.96)		
Living Arrangement						F=12.67	0.053
Living alone	3 (8.11)	2 (2.86)	9 (15.25)	9 (6.67)	50 (9.80)		
Living with family	32 (86.49)	68 (97.14)	50 (84.75)	125 (92.59)	460 (90.20)		
Other	2 (5.41)	0 (.00)	0 (.00)	1 (.74)	0 (.00)		
Primary Caregiver						F=13.0885	0.073
Self	17 (45.95)	31 (44.29)	27 (45.76)	53 (39.26)	25 (49.02)		
Family member	19 (51.35)	39 (55.71)	26 (44.07)	79 (58.52)	26 (50.98)		
Other	1 (2.70)	0 (.00)	6 (10.17)	3 (2.22)	.0 (.00)		
Education Level						H=13.186	0.010
Primary school or below	1 (2.70)	3 (4.29)	2 (3.39)	3 (2.22)	2 (3.92)		
Junior high school	10 (27.03)	19 (27.14)	12 (20.34)	28 (20.74)	7 (13.73)		
Senior high school	19 (51.35)	30 (42.86)	19 (32.20)	41 (30.37)	19 (37.25)		
College	7 (18.92)	17 (24.29)	23 (38.98)	58 (42.96)	20 (39.22)		
Master’s degree or above	0 (.00)	1 (1.43)	3 (5.08)	5 (3.70)	3 (5.88)		
Employment Status						F=28.085	0.003
Full-time employed	2 (5.41)	9 (12.86)	15 (25.42)	33 (24.44)	10 (19.61)		
Resigned	1 (2.70)	2 (2.86)	5 (8.47)	6 (4.44)	5 (9.80)		
Retired/Pensioned	31 (83.78)	43 (61.43)	30 (50.85)	84 (62.22)	24 (47.06)		
Unemployed	3 (8.11)	16 (22.86)	9 (15.25)	12 (8.89)	12 (23.53)		
Healthcare Payment Method						F=19.823	0.084
Employee Medical Insurance	28 (75.68)	52 (74.29)	46 (77.97)	111 (82.22)	36 (70.59)		
Urban Resident Medical Insurance	80 (21.62)	13 (18.57)	10 (16.95)	23 (17.04)	10 (19.61)		
Rural Cooperative Medical Scheme	0 (.00)	1 (1.43)	2 (3.39)	0 (0.00)	0 (3.92)		
Commercial Insurance	1 (2.70)	0 (.00)	0 (1.69)	0 (0.00)	2 (3.92)		
Other	0 (.00)	4.0 (5.71)0	0 (.00)	1 (.74)	1 (1.96)		
Financial Burden of Healthcare Costs						H=7.890	0.096
Very mild	3 (8.11)	3 (4.29)	4 (6.78)	12 (8.89)	8 (15.69)		
Mild	8 (21.62)	24 (34.29)	24 (40.68)	49 (36.30)	19 (37.25)		
Moderate	11 (29.73)	25 (35.71)	16 (27.12)	51 (37.78)	12 (23.53)		
Severe	10 (27.03)	15 (21.43)	11 (18.64)	19 (14.07)	6 (11.76)		
Very severe	5 (13.51)	3 (4.29)	4 (6.78)	4 (2.96)	6 (11.76)		
Primary Etiology of Kidney Disease						F=25.564	0.316
Chronic Glomerulonephritis	13 (35.14)	18 (25.71)	19 (32.20)	44 (32.59)	19 (37.25)		
Diabetic Nephropathy	3 (8.11)	12 (17.14)	5 (8.47)	19 (14.07)	6 (11.76)		
Hypertensive Nephropathy	8 (21.62)	11 (15.71)	10 (16.95)	30 (22.22)	7 (13.73)		
Polycystic Kidney Disease	0 (.00)	1 (1.43)	0 (.00)	1 (0.74)	0 (0.00)		
Hereditary Nephropathy	5 (13.51)	12 (17.14)	2 (3.39)	16 (11.85)	9 (17.65)		
Idiopathic	2 (5.41)	9 (12.86)	13 (22.03)	15 (11.11)	5 (9.80)		
Other	6 (16.22)	7 (10.00)	10 (16.95)	10 (7.41)	5 (9.80)		
Other Chronic Kidney Diseases						χ² =5.592	0.232
Yes	21 (56.76)	31 (44.29)	27 (45.76)	78 (57.78)	23 (45.10)		
No	16 (43.24)	29 (55.71)	32 (54.24)	57 (42.22)	28 (54.90)		
Duration of hemodialysis						H=3.358	0.501
	8.51 ± 1.18	7.95 ± 0.90	8.01 ± 0.89	7.40 ± 0, 57	5.86 ± 0.73		
Hemodialysis Complications						χ² =0.792	0.939
Yes	12 (32.43)	21 (30.00)	17 (28.81)	36 (26.67)	13 (25.49)		
No	25 (67.57)	49 (70.00)	42 (71.19)	99 (73.33)	37 (74.51)		
History of Kidney Transplantation						F=2.786	0.589
Yes	5 (13.51)	7 (10.00)	5 (8.47)	12 (8.89)	2 (3.92)		
No	32 (86.49)	63 (90.00)	54 (91.53)	123 (91.11)	49 (96.08)		
Kidney Transplant Willingness						χ² =8.116	0.422
None	23 (62.16)	34 (48.57)	29 (49.15)	65 (48.15)	18 (35.29)		
Moderate	8 (21.62)	27 (38.57)	19 (32.20)	49 (36.30)	22 (43.14)		
Strong	6 (16.22)	9 (12.86)	11 (18.64)	21 (15.56)	11 (21.57)		
Self-perceived Disease Severity						H=7.108	0.130
Very mild	0 (.00)	0 (.00)	1 (1.69)	3 (2.22)	1 (1.96)		
Mild	4 (10.81)	8 (11.43)	13 (22.03)	23 (17.04)	6 (11.76)		
Moderate	17 (45.95)	40 (57.14)	33 (55.93)	63 (46.67)	29 (56.86)		
Severe	11 (29.73)	19 (27.14)	8 (13.56)	41 (30.37)	10 (19.61)		
Very severe	5 (13.51)	3 (4.29)	4 (6.78)	5 (3.70)	5 (9.80)		
Knowledge Level of Hemodialysis						H=23.979	0.053
Very familiar	2 (5.41)	4 (5.71)	6 (10.17)	11 (8.15)	5 (9.80)		
Fairly familiar	10 (27.03)	24 (34.29)	26 (44.07)	51 (37.78)	22 (43.14)		
Partially familiar	20 (54.05)	21 (30.00)	22 (37.29)	55 (40.74)	18 (35.29)		
Slightly familiar	5 (13.51)	17 (24.29)	4 (6.78)	18 (13.33)	4 (7.84)		
Not familiar at all	0 (.00)	4 (5.71)	1 (1.69)	0 (.00)	2 (3.92)		

H, Kruskal-Wallis H test; F, Fisher’s exact test; χ², Chi-square test.

## 4 Discussion

Using decision tree analysis, this study uncovered distinct psychological profiles underlying BF in MHD patients. Self-efficacy and social support, as core psychological resources, interacted to define five subgroups with markedly different BF levels. Self-efficacy was the dominant predictor, while social support exerted a conditional moderating effect, particularly at moderate self-efficacy levels. Demographic differences in sex, education, and employment across subgroups underscore how social context shapes the configuration of psychological resources. These findings reveal actionable targets for precision psychosocial interventions.

The decision tree model explained 27.8% of the variance in BF among MHD patients, which represents a moderate but clinically meaningful level of predictive power. The remaining unexplained variance likely reflects the complex, multidimensional nature of BF, which is influenced by numerous factors beyond self-efficacy and social support, including personality traits, coping strategies, and cultural factors that were not captured in the current model.

Patients undergoing MHD in this study exhibited moderate-to-high levels of BF, consistent with previous studies (41–43). This suggests that despite the burden of long-term illness, many patients are still able to achieve positive psychological transformation throughout the treatment process. Their capacity for BF may stem from the reappraisal of life circumstances and reconstruction of illness meaning developed over years of dialysis. Decision tree analysis further revealed substantial interindividual variation in BF, resulting in five subgroups with distinct psychological resource profiles. This heterogeneity indicates that BF is not a universal or automatic psychological outcome, but a dynamic construct shaped by the course of illness, individual characteristics, and modulating psychosocial resources—echoing the findings of Prikken et al. (44). Some patients, despite undergoing dialysis for many years, reported persistently low levels of BF, suggesting potential difficulties in emotional regulation or self-reconstruction. These findings support the “individual difference perspective” in psychological nursing, which emphasizes the modulatory role of personal traits in the process of coping with illness (45). Affective temperaments, as stable biological predispositions, may be a significant factor contributing to this heterogeneity. Favaretto et al. (46) demonstrated that affective temperaments significantly influence individual emotional regulation and adaptation patterns, with different temperamental profiles associated with varying levels of sleep quality, treatment adherence, and emotional stability. These temperamental characteristics may explain why patients exhibit varying psychological resource configurations and BF levels despite similar disease and treatment circumstances. Future research integrating affective temperament assessment could provide deeper insights into the neurobiological underpinnings of positive psychological adaptation in chronic kidney disease patients.

The decision tree model identified self-efficacy as the primary variable distinguishing BF levels, consistent with its central role in social cognitive theory. As discussed by Barlattani et al., within the social cognitive model, self-efficacy is a key construct influencing health-related behaviors and psychological adaptation, and is regarded as a major predictor of both intention and health behavior (47). Patients in the “Low Self-Efficacy Constrained Group” exhibited significantly lower BF compared to other groups. Notably, in the “Internally Belief Driven Group” and “Balanced Resource Adaptation Group” some individuals maintained moderate-to-high BF scores even with only moderate levels of social support. This underscores the critical influence of efficacy beliefs on the capacity to ascribe positive meaning to illness experiences. This finding aligns with Bandura’s self-efficacy theory (15), which posits that belief in one’s ability to cope with challenges influences emotional regulation, coping behavior, and goal persistence. High self-efficacy enhances patients’ confidence in managing symptoms and treatment, thereby alleviating stress and promoting positive adaptation (48). Conversely, low self-efficacy is associated with emotional distress and cognitive helplessness, hindering the recognition of benefits. There BF should prioritize improving self-efficacy through structured cognitive restructuring, role modeling, and verbal encouragement.

Although social support was not the primary splitting variable in the model, its synergistic role was most evident in the “ Balanced Resource Adaptation Group” and “Psychological Resource Integrated Advantage Group”. Patients with high levels of self-efficacy and social support achieved the highest BF scores. Bland (49) reported that “frequent contact with friends and family during social isolation was associated with more positive emotional processing and cooperative behaviors,” which aligns with our observations in hemodialysis patients—social support functioning as a critical catalyst for positive psychological adaptation. McKeown et al (50)demonstrated that social connections are essential for maintaining positive cognitive processing. For dialysis patients, the social limitations imposed by treatment requirements may parallel the effects of isolation measures, potentially weakening social connections and impairing their capacity for positive meaning construction. Patients in these groups, with high levels of both GES and PSSS, achieved the highest BF scores. Social support functions through mechanisms such as emotional reassurance, informational guidance, tangible assistance, and strengthened sense of belonging, all of which facilitate the construction of positive illness meaning and reinforce adaptive confidence and hope (51, 52). Existing research highlights the role of social support as a critical external resource in chronic illness, capable of buffering stress, promoting adaptive responses, regulating emotion, and fostering psychological growth and benefit recognition (53, 54). For patients in the “Internally Belief Driven Group” although BF was not entirely suppressed, the lack of external support likely limited the depth and breadth of psychological growth. These findings suggest that both internal and external psychological resources must be strengthened in parallel. Strategies such as enhancing family support, fostering patient–provider trust, and encouraging peer support networks can provide a robust foundation for BF development (55–57).

Notable sociodemographic differences were observed across subgroups, suggesting that psychological resource configurations may be shaped by underlying demographic factors. In terms of gender distribution, both male and female patients were primarily represented in the “Balanced Resource Adaptation Group,” characterized by moderately high self-efficacy and strong social support. This indicates that balanced psychological resources—regardless of gender—can facilitate better BF outcomes. However, a notably higher proportion of female patients appeared in the “Psychological Resource-Integrated Advantage Group,” suggesting that when women possess both high self-efficacy and high social support, they are more likely to achieve optimal BF. “Statistical analysis confirmed this gender-related pattern (χ² = 9.72, *p* = 0.05). Column proportion tests revealed that the proportion of females in the “Psychological Resource Integration Advantage” subgroup (Subgroup 5, 60.78%) was significantly higher than in the “Internal Belief-Driven” subgroup (Subgroup 3, 33.90%) and the “Balanced Resource Adaptation” subgroup (Subgroup 4, 42.22%). This gender effect may reflect women’s strengths in emotional expression, social network maintenance, and help-seeking behavior (58). Education level showed a significant association with subgroup membership(*p*=0.010). Over 45% of patients with a college degree or higher were concentrated in Subgroups 4 and 5, whereas only 18.92% of such patients were found in Subgroup 1. Higher educational attainment may contribute to better health literacy and problem-solving capacity, enabling individuals to recognize internal resources, utilize social support more effectively, and develop deeper levels of BF (15, 47). Regarding employment status, retired or unemployed individuals were more prevalent across all subgroups, especially in Subgroups 1 and 2. In contrast, Subgroups 3 and 5 had higher proportions of patients with current or previous full-time employment. These findings suggest a close relationship between employment and psychological resource reserves. Long-term unemployment or retirement may result in loss of social roles, weakened social connections, and reduced sense of control, ultimately impairing self-efficacy development and support network maintenance—leading to lower BF levels (59). Therefore, psychological interventions should account for patients’ sociodemographic profiles and be tailored accordingly to optimize BF outcomes and enhance intervention precision.

## 5 Limitations

This study employed a cross-sectional design with samples drawn from five tertiary hospitals in the metropolitan area of Shanghai, eastern China, providing reasonable representation of urban hemodialysis patients. However, this sampling approach introduced a distinct urban bias, potentially limiting the generalizability of findings to community or rural dialysis centers. Future research should broaden sample coverage by including more diverse geographic regions and healthcare facility levels to enhance external validity. The study focused solely on self-efficacy and social support as predictor variables. The limited explanatory power of the model suggests that BF may be influenced by unmeasured confounders such as individual affective temperament, depression, coping strategies, social desirability bias, and health literacy. Incorporating a wider range of relevant variables is recommended to develop a more comprehensive predictive model. Data collection relied entirely on self-report questionnaires, which may introduce social desirability bias, particularly in measures of BF and social support. Future studies should consider multi-method data collection approaches, including objective assessments, clinical observations, or qualitative interviews, to minimize methodological bias.Although cross-validation was used to evaluate the stability of the decision tree model, the absence of external validation samples or more rigorous techniques such as bootstrapping may increase the risk of overfitting, potentially limiting the generalizability of the findings. Furthermore, the cross-sectional design precludes the examination of dynamic changes in BF or causal relationships among variables. While CART analysis can identify predictive relationships and interaction patterns, these should not be interpreted as causal. Longitudinal studies are needed to elucidate the developmental trajectories of BF and its dynamic associations with psychological resources.

## 6 Implications and practical significance

Our findings demonstrate that BF is a multifaceted construct shaped by the interaction of multiple psychological resources. Using a decision tree model based on self-efficacy and social support, we identified five subgroups with distinct profiles, offering a foundation for stratified psychological intervention. Patients with limited psychological resources should be prioritized for foundational support targeting coping beliefs, social connectedness, and psychoeducation. Conversely, those with relatively strong resources may benefit from advanced strategies focused on deepening self-reflection, expanding positive emotions, and enhancing meaning-making. Traditional “one-size-fits-all” approaches may fail to meet the heterogeneous psychological needs of dialysis patients. Instead, precision interventions grounded in psychological resource structures may enhance BF, improve quality of life, and support long-term adherence—advancing the clinical application of targeted psychological care in chronic disease management.

## 7 Conclusion

This study employed decision tree methodology to reveal the complex heterogeneity in BF among MHD patients. Results indicate that self-efficacy and social support are key psychological resources influencing BF, demonstrating diverse pathways through which patients cope with chronic illness challenges. For patients with low self-efficacy, clinical teams should prioritize self-efficacy enhancement programs, such as progressive self-management training and successful experience accumulation. For patients with higher self-efficacy but insufficient social support, interventions should focus on strengthening social support networks, including family support training, peer support groups, and community resource connections. For patients with robust psychological resources, maintenance psychological interventions and empowerment programs may be appropriate. Healthcare teams can utilize the decision tree classification tool proposed in this study for rapid screening and grouping, enabling optimized resource allocation and precise intervention matching. Future research should employ longitudinal designs to explore the dynamic development of BF, incorporate additional potential influencing factors to assess applicability across different cultural and clinical contexts, and develop differentiated intervention protocols for various psychological subgroups to validate their effectiveness in improving psychological adaptation and quality of life among dialysis patients.

## Data Availability

The original contributions presented in the study are included in the article/**Supplementary Material**. Further inquiries can be directed to the corresponding authors.
