# Supplementary material for: Heterogeneity of benefit finding in maintenance hemodialysis patients: a decision tree-based subgroup analysis of self-efficacy and social support
Source: Front Psychiatry. 2025 Sep 18;16:1665458. doi: 10.3389/fpsyt.2025.1665458 (PMC12488675; doi:10.3389/fpsyt.2025.1665458)
Supplement: Supplementary file 1 [file DataSheet1.pdf]

Part 1: Data Preprocessing

Load dataset containing Benefit Finding Scale (BFS), General Self-Efficacy Scale (GSES), and Perceived Social Support Scale (PSSS)

Check and handle missing values

Extract feature matrix X (GSES, PSSS) and target variable y (BFS)

Standardize feature matrix using StandardScaler, resulting in X_std

X_std = (X - mean(X)) / std(X) # Z-score standardization

Part 2: Decision Tree Subgroup Identification

Key parameter settings:

- criterion = squared_error
- max_depth = 3
- min_samples_leaf = 0.1*n
- random_state = 42
- Initialize Decision Tree Regressor model with above parameters
- Train decision tree using feature matrix X and target variable y: tree.fit(X, y)
- Obtain leaf node identifiers for each sample: leaf_ids = tree.apply(X)
- Add leaf node identifiers as subgroup labels to the original dataset

Part 3: Model Validation and Evaluation

1. Evaluate decision tree model using 5-fold cross-validation

- Partition data using KFold (n_splits=5, shuffle=True, random_state=42)
- Calculate R^2 score for each fold
- Compute average R^2 score and standard deviation

2. Calculate overall model performance metrics

- R^2 score (coefficient of determination): r2_score(y, y_pred)
- Mean Squared Error (MSE): mean_squared_error(y, y_pred)
- Mean Absolute Error (MAE): mean_absolute_error(y, y_pred)
- Explained Variance Score: explained_variance_score(y, y_pred)

Part 4: Subgroup Visualization and Feature Analysis

1. Plot decision tree structure showing decision rules and node information
2. Create 2D scatter plot displaying subgroup distribution based on GSES and PSSS
3. Calculate mean BFS, GSES, and PSSS scores for each subgroup
4. Generate radar chart comparing distribution of the three psychological resources across subgroups

Core Algorithm Python Code Snippets

Decision tree model definition and fitting:

```
min_samples_leaf = int(0.1 * len(df)) # 10% of total sample
tree = DecisionTreeRegressor(
criterion='squared_error',
max_depth=3,
min_samples_leaf=min_samples_leaf,
```

```
random_state=42
)
tree.fit(X, y)
```

Cross-validation code:

```
kf = KFold(n_splits=5, shuffle=True, random_state=42)
cv_scores = cross_val_score(tree, X, y, cv=kf, scoring='r2')
```
