# Supplementary material for: Heterogeneity of benefit finding in maintenance hemodialysis patients: a decision tree-based subgroup analysis of self-efficacy and social support
Source: Front Psychiatry. 2025 Sep 18;16:1665458. doi: 10.3389/fpsyt.2025.1665458 (PMC12488675; doi:10.3389/fpsyt.2025.1665458)
Supplement: Supplementary file 2 [file DataSheet2.pdf]

Appendix B: Complete Items of the Benefit Finding Scale (BFS)

Instructions: This questionnaire focuses on the positive changes in your thoughts and behaviors as a result of your illness. Please read each statement carefully. If you do not have any positive thoughts or actions in the statement, please mark "√" on "none". If you often think of or do the content in the statement, please mark "√" on "a lot". Thank you for your cooperation!

Dimension	Item	Not at all	Slightly	Moderately	Quite a bit	Very much
Spiritual growth	1. I understand that everyone must face death eventually, and I am no longer afraid.	0	1	2	3	4
	2. I believe that everyone's life experiences have meaning, and my current illness is also an experience.	0	1	2	3	4
Appreciation of living and life	3. I have learned to appreciate life and discover the beauty of living.	0	1	2	3	4
	4. I realize that life is finite, and time is precious.	0	1	2	3	4
	5. I recognize that I cannot always know what will happen tomorrow, so I need to live each day well.	0	1	2	3	4
	6. I feel grateful for every day of my life.	0	1	2	3	4
	7. I realize that health and life are the most important things.	0	1	2	3	4
Awareness of social support	8. I realize that my family and friends care about me very much.	0	1	2	3	4
	9. I realize that I can rely on my family and friends for help.	0	1	2	3	4
	10. My relationship with my family has become more intimate and harmonious.	0	1	2	3	4
	11. I can obtain concern and assistance from the community and related organizations.	0	1	2	3	4
Personal growth	12. I have become more optimistic and can view problems in a more positive way.	0	1	2	3	4
	13. I have adjusted my	0	1	2	3	4

	mindset to reduce the distress caused by my illness.					
	14. I can adapt to changes in my circumstances.	0	1	2	3	4
	15. I find myself stronger than I imagined, able to cope with pressure and difficulties.	0	1	2	3	4
	16. I have learned to control my emotions and no longer get upset over trivial matters.	0	1	2	3	4
	17. I have become more responsible.	0	1	2	3	4
	18. I am more willing to express my inner thoughts and feelings.	0	1	2	3	4
Altruistic behavior	19. I will share relevant information about my treatment with those who need it.	0	1	2	3	4
	20. I will encourage patients with similar diseases as mine.	0	1	2	3	4
	21. I will assist patients with similar diseases as mine.	0	1	2	3	4
Health behavior changes	22. I have started adopting a healthier lifestyle.	0	1	2	3	4
	23. I have begun cultivating regular work habits.	0	1	2	3	4
	24. I have started paying attention to my diet and eating more healthily.	0	1	2	3	4
	25. I have begun engaging in appropriate physical exercise.	0	1	2	3	4
	26. I have tried to quit some bad habits, such as smoking and drinking.	0	1	2	3	4

Note： A 5-point Likert scale was used, from "none at all" to "very much", and 0 points were scored in turn. The total score ranged from 0 to 104, with higher scores indicating a higher level of benefit finding experienced by patients.

Statistical processing of questionnaire preparation: Excel 2016 was used to input and check the data, and IBM SPSS Statistics 21.0 was used for statistical analysis. According to the purpose of the study, the item analysis, validity test and reliability test were carried out as the basis for the preparation of the formal questionnaire.

Results of the pre-survey: 1. General characteristics of the pre-survey subjects The research subjects involved two tertiary hospitals and four internal medicine departments (neurology, cardiology, respiratory medicine and endocrinology). A total of 380 questionnaires were distributed and 338 were returned, with a recovery rate of 88.9%.

1. Item analysis: ***P*** of each item in the questionnaire was <0.05,Critical Ratio(CR) value reached the significant level, and the item discrimination was good, as shown in Table A1.

TableA1: Analysis Results of the BFS

Item Number	Total Score ≤74 (N=87)	Total Score ≥97 (N=93)	CR Value	P
1	1.66±1.139	2.96±1.052	7.970	<0.001
2	1.90±1.303	3.06±1.009	6.692	<0.001
3	2.70±0.954	3.90±0.297	11.256	<0.001
4	2.97±0.970	3.94±0.385	8.709	<0.001
5	2.77±0.997	3.70±0.763	6.986	<0.001
6	2.77±0.973	3.90±0.419	10.029	<0.001
7	1.55±1.265	2.83±1.299	6.672	<0.001
8	3.01±0.982	3.96±0.204	8.802	<0.001
9	2.07±1.043	3.41±0.924	9.135	<0.001
10	1.77±1.042	3.61±0.590	14.464	<0.001
11	1.89±1.061	3.34±1.108	9.009	<0.001
12	2.57±1.207	3.82±0.551	8.785	<0.001
13	2.10±1.100	3.75±0.503	12.788	<0.001
14	1.99±1.029	3.74±0.509	14.344	<0.001
15	2.03±0.841	3.72±0.497	16.226	<0.001
16	1.90±1.046	3.66±0.542	14.029	<0.001
17	1.99±0.869	3.58±0.596	14.413	<0.001
18	1.93±1.009	3.54±0.600	12.873	<0.001
19	2.32±0.982	3.75±0.458	12.384	<0.001
20	1.83±1.059	3.51±0.636	12.778	<0.001
21	2.51±1.160	2.86±1.069	2.133	<0.05
22	1.97±1.050	3.82±0.416	15.356	<0.001
23	1.94±1.103	3.85±0.360	15.374	<0.001

Item Number	Total Score ≤74 (N=87)	Total Score ≥97 (N=93)	CR Value	P
24	1.76±1.067	3.77±0.513	15.972	<0.001
25	2.05±0.975	3.68±0.574	13.558	<0.001
26	2.14±1.002	3.80±0.431	14.246	<0.001
27	2.17±0.943	3.78±0.463	14.414	<0.001
28	1.97±1.005	3.55±0.651	12.617	<0.001
29	1.94±1.417	3.27±1.336	6.463	<0.001

2. Construct validity: exploratory factor analysis, KMO and Bartlett's spherical test results (Table A2); The percentage of explained variance for the final 6-factor structure (Table A3) The final rotated factor loading matrix (TableA4)

Table A2: KMO and Bartlett's Sphericity Test for BFS

Measure	Value
KMO Sampling Adequacy	0.915
Bartlett's Test of Sphericity	
- Approximate Chi-Square	4639.206
- Degrees of Freedom	325
- Significance	.000

Table A3: Total Variance Explained

Factor	Initial Eigenvalues	Extraction Sums of Squared Loadings	Rotation Sums of Squared Loadings
	Total	% of Variance	Cumulative %
1	9.780	37.614	37.614
2	2.104	8.093	45.707
3	1.679	6.459	52.166
4	1.656	6.369	58.535
5	1.150	4.422	62.956
6	1.015	3.903	66.859

Table A4: Factor Loading Matrix for Each Item

Item	Component 1	Component 2	Component 3	Component 4	Component 5	Component 6
BF16	.853	.081	.119	.052	.072	.076
BF14	.765	.149	.204	.178	.142	-.005
BF17	.739	.231	.134	.086	.099	.167
BF15	.716	.080	.126	.159	.185	.214
BF18	.678	.198	.106	.027	.224	.170
BF13	.593	.264	.297	.376	.020	-.069
BF20	.577	.315	.140	.139	.229	-.001
BF26	.255	.775	.127	.120	.220	.034
BF28	.264	.750	.157	.115	.081	.091
BF27	.380	.732	.098	.177	.080	-.048
BF25	.323	.707	.118	.138	.270	.086
BF29	-.067	.488	.663	.105	.153	.085
BF04	.180	.101	.847	.066	.154	.002
BF05	.029	.094	.826	.036	.051	.091
BF06	.170	.096	.825	.156	.161	.047
BF03	.310	.107	.652	.241	.075	.033
BF08	.235	.266	.507	.306	.181	-.083
BF11	.028	.036	.060	.760	.217	.169
BF12	.226	.171	.251	.737	-.005	.033
BF10	.296	.229	.067	.649	.200	.035
BF09	.049	.191	.194	.563	.041	.280
BF23	.254	.244	.241	.220	.781	.001
BF24	.281	.318	.212	.146	.774	.064
BF22	.292	.375	.155	.144	.687	.142
BF02	.132	.031	.039	.117	.073	.821
BF01	.187	.127	.029	.188	.029	.789

3.Reliability (1) Internal consistency Cronbach a coefficient of the internal consistency reliability of the questionnaire was measured according to the results of the pre-survey. The Cronbach'a coefficients of the BFS are shown in Table A5.

TableA5: Cronbach's Alpha Coefficients for BFS Dimensions and Overall

Questionnaire	Dimension	Number of Items	Cronbach's Alpha
BFS	/	26	0.924
	Personal Growth	7	0.893
	Health Behavior Change	5	0.800
	Appreciation of Life and Living	5	0.855
	Understanding Social Support	4	0.747
	Prosocial Behavior	3	0.892
	Spiritual Growth	2	0.664

(2) Test-retest reliability (Table A6)

Table A6: Test-Retest Reliability for BFS Dimensions and Overall

Questionnaire	Dimension	Number of Items	Test-Retest Reliability
BFS	/	26	0.902
	Personal Growth	7	0.868
	Health Behavior Change	5	0.873
	Appreciation of Life and Living	5	0.783
	Understanding Social Support	4	0.856
	Prosocial Behavior	3	0.844
	Spiritual Growth	2	0.847
